# Biomolecular changes and subsequent time-dependent recovery in hippocampal tissue after experimental mild traumatic brain injury

**DOI:** 10.1038/s41598-021-92015-3

**Published:** 2021-06-14

**Authors:** Sebnem Garip Ustaoglu, Mohamed H. M. Ali, Fazle Rakib, Erwin L. A. Blezer, Caroline L. Van Heijningen, Rick M. Dijkhuizen, Feride Severcan

**Affiliations:** 1 Department of Medical Biochemistry, Faculty of Medicine, Altinbas University, Bakirkoy, Istanbul Turkey; 2 Diabetes Research Center, Qatar Biomedical Research Institute (QBRI), Hamad Bin Khalifa University (HBKU), Qatar Foundation (QF), P.O. Box 34110, Doha, Qatar; 3 Department of Chemistry and Earth Sciences, Qatar University, Doha, Qatar; 4 Biomedical MR Imaging and Spectroscopy Group, Center for Image Sciences, University Medical Center Utrecht, Utrecht, The Netherlands; 5 Department of Biophysics, Faculty of Medicine, Altinbas University, Bakirkoy, Istanbul Turkey; 6 Department of Biological Sciences, Middle East Technical University, Ankara, Turkey

**Keywords:** Biophysical chemistry, Brain injuries

## Abstract

Traumatic brain injury (TBI) is the main cause of disability and mortality in individuals under the age of 45 years. Elucidation of the molecular and structural alterations in brain tissue due to TBI is crucial to understand secondary and long-term effects after traumatic brain injury, and to develop and apply the correct therapies. In the current study, the molecular effects of TBI were investigated in rat brain at 24 h and 1 month after the injury to determine acute and chronic effects, respectively by Fourier transform infrared imaging. This study reports the time-dependent contextual and structural effects of TBI on hippocampal brain tissue. A mild form of TBI was induced in 11-week old male Sprague Dawley rats by weight drop. Band area and intensity ratios, band frequency and bandwidth values of specific spectral bands showed that TBI causes significant structural and contextual global changes including decrease in carbonyl content, unsaturated lipid content, lipid acyl chain length, membrane lipid order, total protein content, lipid/protein ratio, besides increase in membrane fluidity with an altered protein secondary structure and metabolic activity in hippocampus 24 h after injury. However, improvement and/or recovery effects in these parameters were observed at one month after TBI.

## Introduction

Traumatic brain injury (TBI), which is the main cause of disability and mortality in individuals under the age of 45 years^1^, is believed to become the third leading cause of death worldwide by 2020^2^. Tissue injury after TBI is associated with changes in neurotransmitters, cytokines, biochemical mediators as well as genetic changes, along with structural and metabolic changes resulting in exitotoxicity, neuroinflammation and cell death^2,3^. The changes in brain function due to TBI, can be temporary or permanent according to the severity of trauma and injury type^1^. The diagnosis of TBI severity is done primarily based on the Glasgow Coma Scale (GCS), which has become a controversial issue. According to GCS, patients are categorized into mild, moderate and severe TBI by evaluating the consciousness, which gives prognostic information without any knowledge about underlying pathologies^4^. Moreover, assessment of brain damage and development of neurological symptoms can offer critical additional information, not provided by the GCS, to direct the clinical management.

Thus, elucidation of the molecular and structural alterations in brain tissue due to TBI is crucial to understand the secondary and long-term effects after TBI, and to develop and apply the correct therapies for rapid recovery.

A great number of techniques have been used for evaluating the alterations in structure and function in brain tissue due to TBI in both clinical and experimental studies. The currently used clinical imaging techniques such as magnetic resonance imaging (MRI) and positron emission tomography (PET), may be supported by emerging technologies including proteomic and metabolomic techniques and optical spectroscopy^5^.

Vibrational spectroscopic techniques including Fourier transform infrared (FTIR) spectroscopy, near infrared spectroscopy (NIRS) and Raman spectroscopy enable quantitative analysis of alterations in biomolecules due to different factors, such as injury, disease state and environmental pollution, by calculating the intensity and/or area of related absorption bands in the spectrum. For this aim, the examination of molecular and structural composition of tissues can be done in unfixed and fixed tissue sections without using additional chemical reagents, unlike traditional histological methods. Previously, NIRS has been used for non-invasive monitoring of cerebral tissue oxygenation and perfusion^6^, and intracerebral, subdural and epidural hematomas in brain tissues after TBI^7,8^. Furthermore, Raman spectroscopy has been applied for measurements in different models of brain injury, such as from radiation^9^, penetrating trauma^10^, peripheral nerve injury^11^ and focal TBI^12^.

Molecular changes in the structure and concentration of biomolecules such as lipids, proteins and carbohydrates can be monitored by using FTIR Imaging data^13–15^. FTIR spectroscopy has been previously used to assess brain tissue status in response to various conditions, including radiation^16^, Alzheimer’s disease^17^, brain tumors^18^, multiple sclerosis^19^, traumatic axonal injury (TAI)^20^, diffuse axonal injury (DAI)^21,22^, ischemic brain injury^23^, and intracerebral hemorrhage^24^. Previous FTIR Spectroscopy and Imaging studies on brain tissues by our group include investigation of the effects of whole body ionizing radiation on rat brain tissue^16^ and homogenate membrane^25^, vitamin A deficiency (VAD) on hippocampal region in rat brain^26^, pentylenetetrazol-induced seizures on homogenate rat brain tissues^27^, ischemic stroke on rat brain^28^, as well as general biochemical analysis of different rat brain regions in healthy^29^ and 3 h post-TBI^30^ animals.

These previous studies indicated the potential of FTIR Imaging to identify molecular changes, such as contextual and structural alterations in lipid and proteins, in brain tissues, which are relevant in brain pathophysiology. The ability to detect the spatial variations in both protein and lipid chemistry makes FTIR Imaging a promising technology for investigating key pathways after TBI at different post-injury time points. In the light of this information, the molecular effects of mild TBI in rat brain were investigated in hippocampal tissue, a region that is significantly affected by TBI, at 24 h and 1 month after the injury by FTIR microspectroscopy to determine the acute and chronic effects of TBI, respectively.

## Results

In the present study, time-dependent molecular changes in the rat hippocampus after experimental TBI were investigated using FTIR Imaging. Acute molecular changes were determined at 24 h after TBI, while chronic changes were examined one month after TBI.

**Figure 1:**
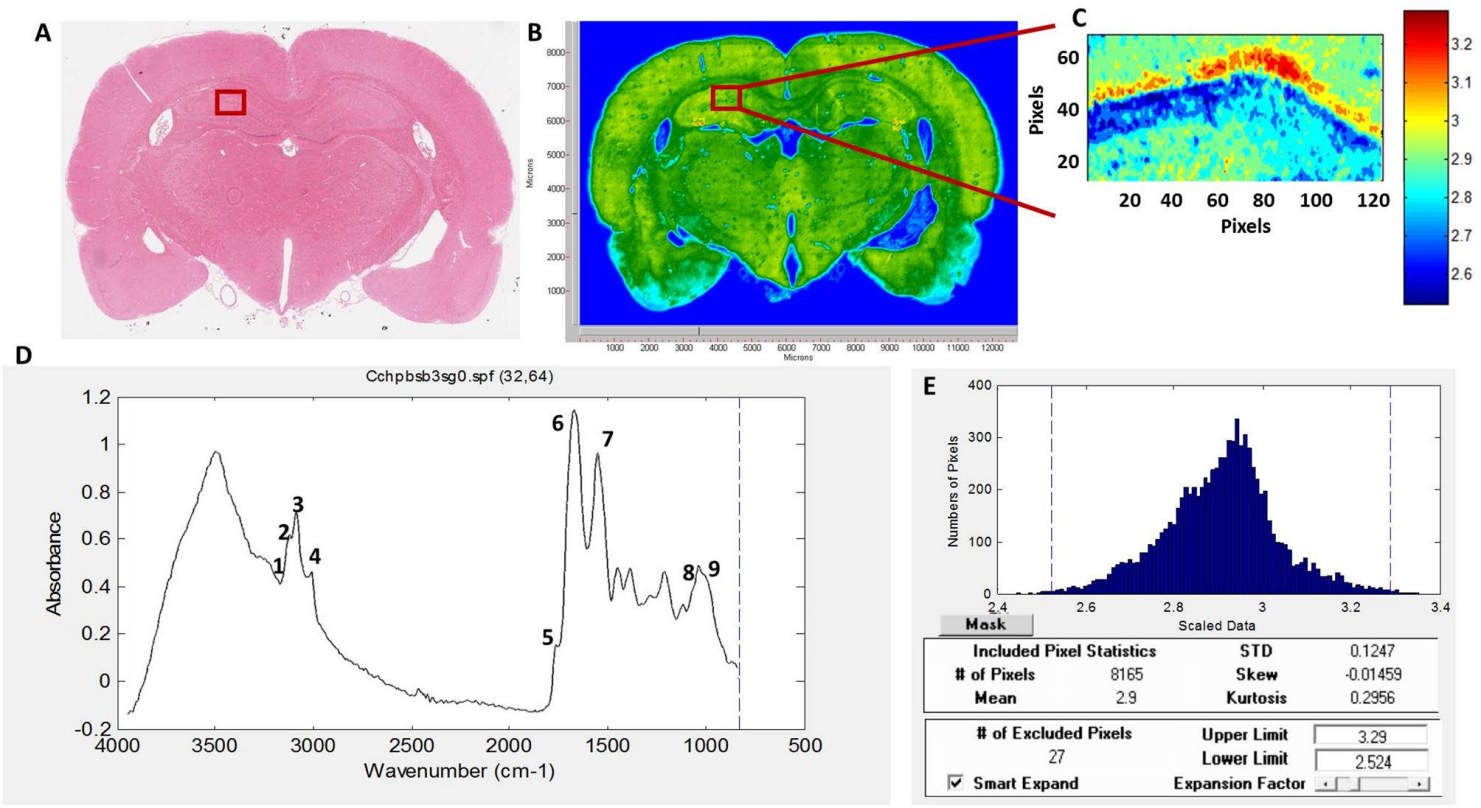
(**A**) Histological image of an H&E stained brain section. (**B**) Representative FTIRI of the rat brain tissue shown in (**A**) with a rectangular label in hippocampal area indicating anatomic site examined by FTIRI. (**C**) Typical FTIR image from the rectangular labeled area before spectral processing. (**D**) The average spectra of hippocampal tissue of the control group in 4000–800 cm^−1^ region (The spectra were normalized with respect to the Amide A band). (**E**) The pixel histogram for the image shown in (**C**).

**Figure 2:**
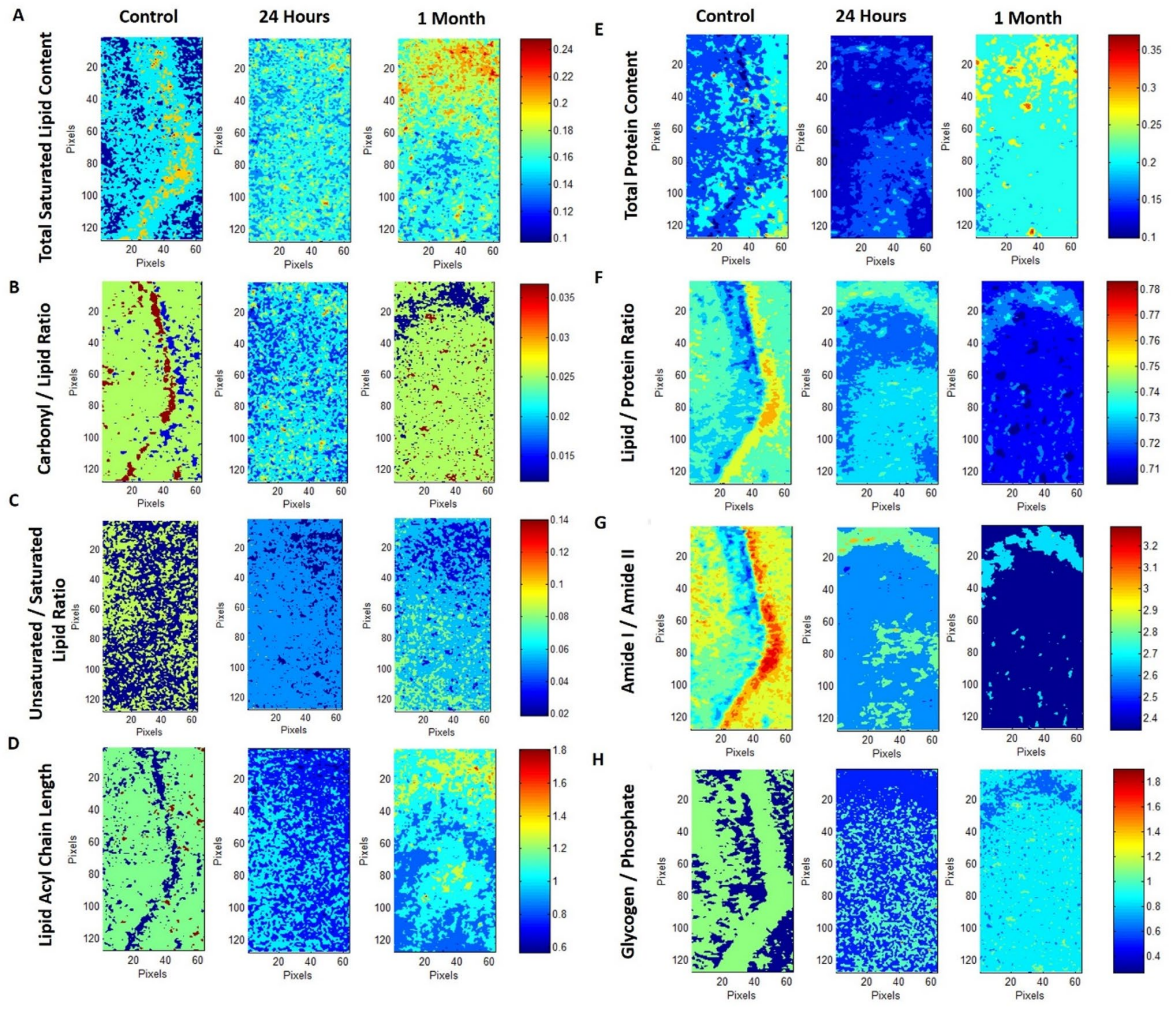
Typical FTIR images of total saturated lipid content (**A**), carbonyl/lipid (**B**), unsaturated/saturated lipid (**C**), lipid acyl chain length (**D**), total protein content (**E**), lipid/protein (**F**), amide I/amide II (**G**) and glycogen/phosphate (**H**) band area ratios in left hippocampal tissue of control, 24 h and 1-month TBI groups. Color bars represent the scales for each of the parameters. Axes are in pixels, where one pixel is 6.5 μm. The pixel histograms for these images are shown in Fig. 3.

**Figure 3:**
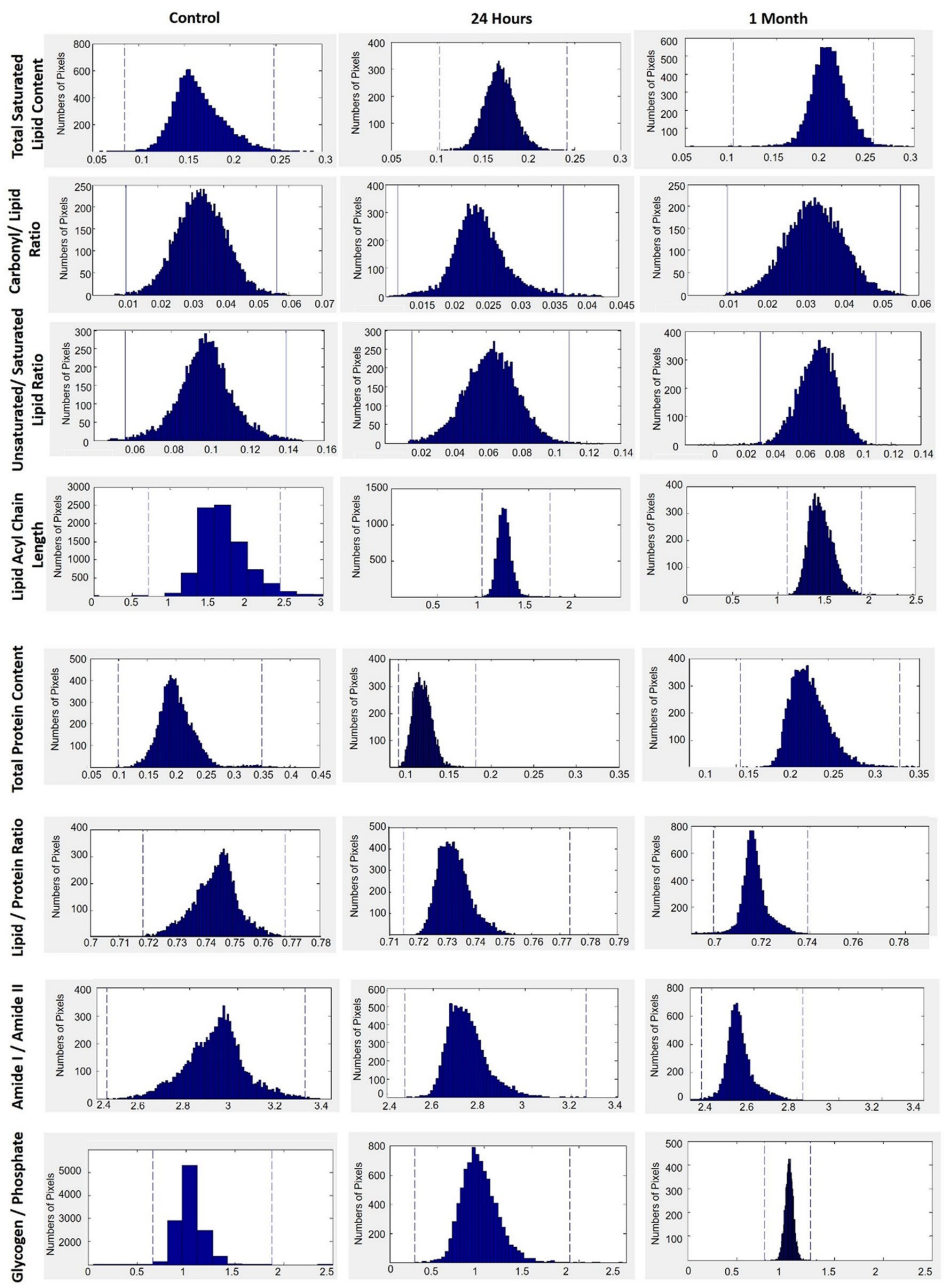
The pixel histograms for the FTIR images of total lipid content, carbonyl/lipid, unsaturated/saturated lipid, lipid acyl chain length, total protein content, lipid/protein, amide I/amide II and glycogen/phosphate ratios shown in Fig. 2 in hippocampal tissue of control, 24 h and 1-month TBI groups.

Representative infrared spectra (4000–500 cm^**−**1^ region) obtained from the hippocampus in healthy rat brain are presented in Fig. 1D. Spectral bands used in the analysis are labelled in the figure and their assignments are given in details in Table 1. Spectral parameters including total protein and lipid content, lipid/protein ratio, the amount of carbonyl groups in lipids, the amount of unsaturation in lipids, lipid acyl chain length, lipid order and membrane fluidity, the structural changes in the tissue proteins and metabolic activity were determined by calculation of infrared integrated band area ratios, band intensity ratios, band frequency and bandwidth values. Representative chemical (absorption) maps for calculated biomolecular parameters using peak area ratios and pixel histograms of these chemical maps are presented in Figs. 2 and 3, respectively. The integrated spectral regions and baseline points for the infrared bands used in this study are given in Table 1S as supplementary material. The differences in section thickness may result in concentration-dependent spectral changes in absorbance values, thus the band area ratios were used for the calculation of contextual and structural parameters instead of using the area of the band itself. By this way, possible errors due to section thickness were avoided in FTIR image analysis^31^.

**Table 1:** The assignments of the bands used in the analysis of the spectra of rat hippocampal brain tissue in 3100–1000 cm^−1^ wavenumber region (Cakmak et al. 2012; 2016, Demir et al., 2015; Ali et al., 2018).

Band #	Wavenumber (cm^−1^)	Definition of the assignment
1	3012	Olefinic = CH: unsaturated lipids
2	2957	CH_3_ antisymmetric stretching: lipids and protein side chains
3	2923	CH_2_ antisymmetric stretching: mainly lipids with little contribution from proteins
4	2852	CH_2_ symmetric stretching: mainly lipids with little contribution from proteins
5	1732	Carbonyl ester stretching: lipids
6	1651	Amide I: proteins (80% protein C=O stretching, 10% protein N–H bending, 10% C–N stretching)
7	1547	Amide II: proteins (60% protein N–H bending, 40% C–N stretching)
8	1075	PO_2_^−^ symmetric stretching: phospholipids
9	1049	νC–O + δC–O stretching band: glycogen

**Figure 4:**
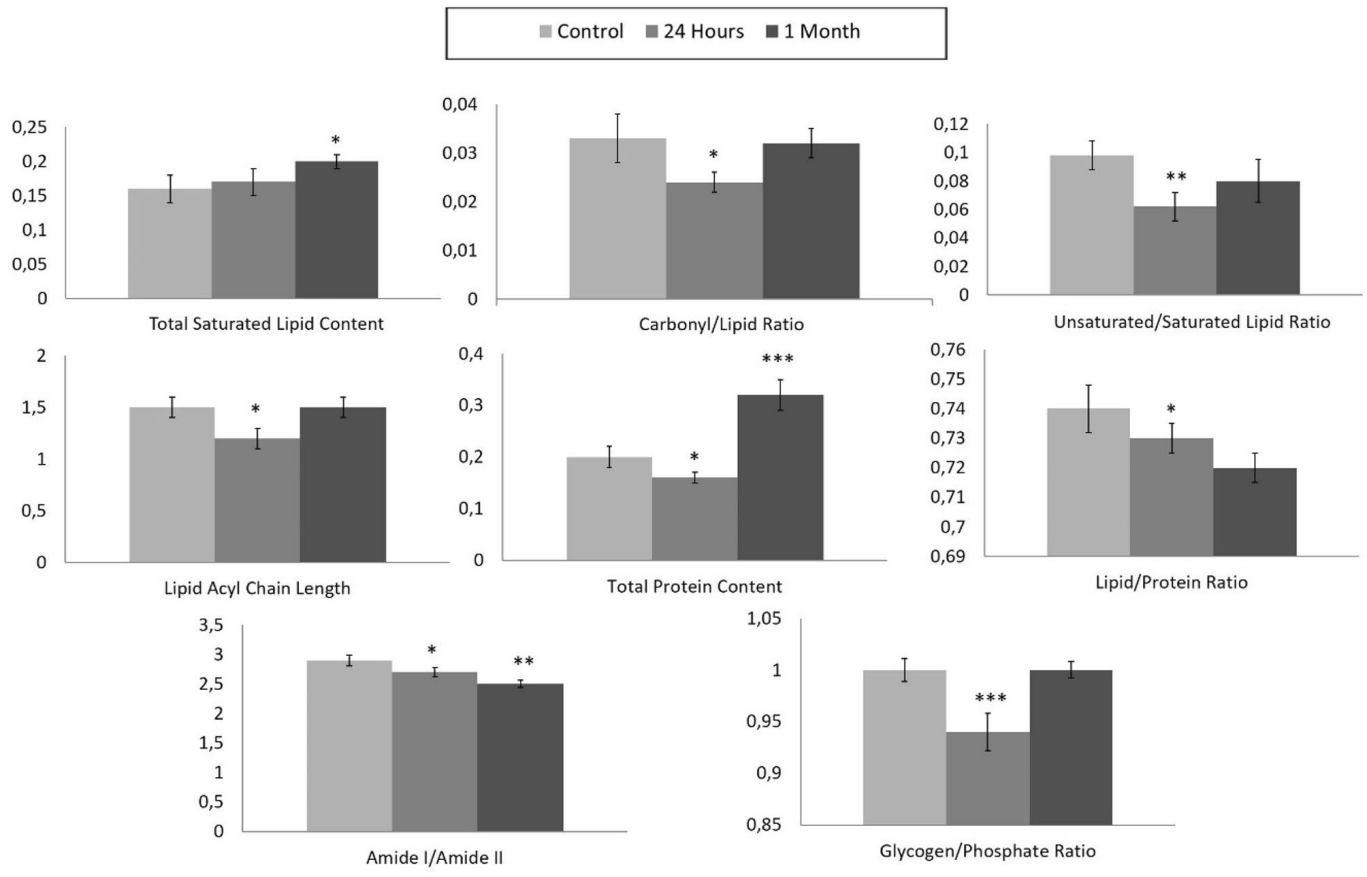
Histograms of the calculated parameter values (total saturated lipid content, carbonyl/lipid, unsaturated/saturated lipid, lipid acyl chain length, total protein content, lipid/protein, amide I/amide II and glycogen/phosphate band area ratios) for 24 h and 1-month TBI groups. Statistical significance was determined by one-way ANOVA with Tukey’s post hoc test and the 95% confidence interval. Bars represent mean ± SD. *p ≤ 0.05; **p ≤ 0.01, ***p ≤ 0.001.

The total saturated lipid amount was calculated by taking the ratio of the integrated area of the CH_2_ anti-symmetric stretching (2944–2896 cm^−1^) band to CH-region (2988–2832 cm^−1^) (Fig. 4). The CH_3_ symmetric stretching band in the CH region is mainly due to proteins, and CH_3_ anti-symmetric stretching band has equal contribution from lipids and proteins^31,32^. However, as these bands occupy a very small area in the CH region, they were ignored in the calculations and the CH region was used as the total saturated lipid region. The total saturated lipid content was increased slightly at 24 h and significantly at 1 month after TBI, as compared to the control group (Figs. 2, 4). The significant increase in lipid content was also clearly seen in the pixel histograms, in which the pixels were moved to the right side of the histogram with higher values (Fig. 3).

**Figure 5:**
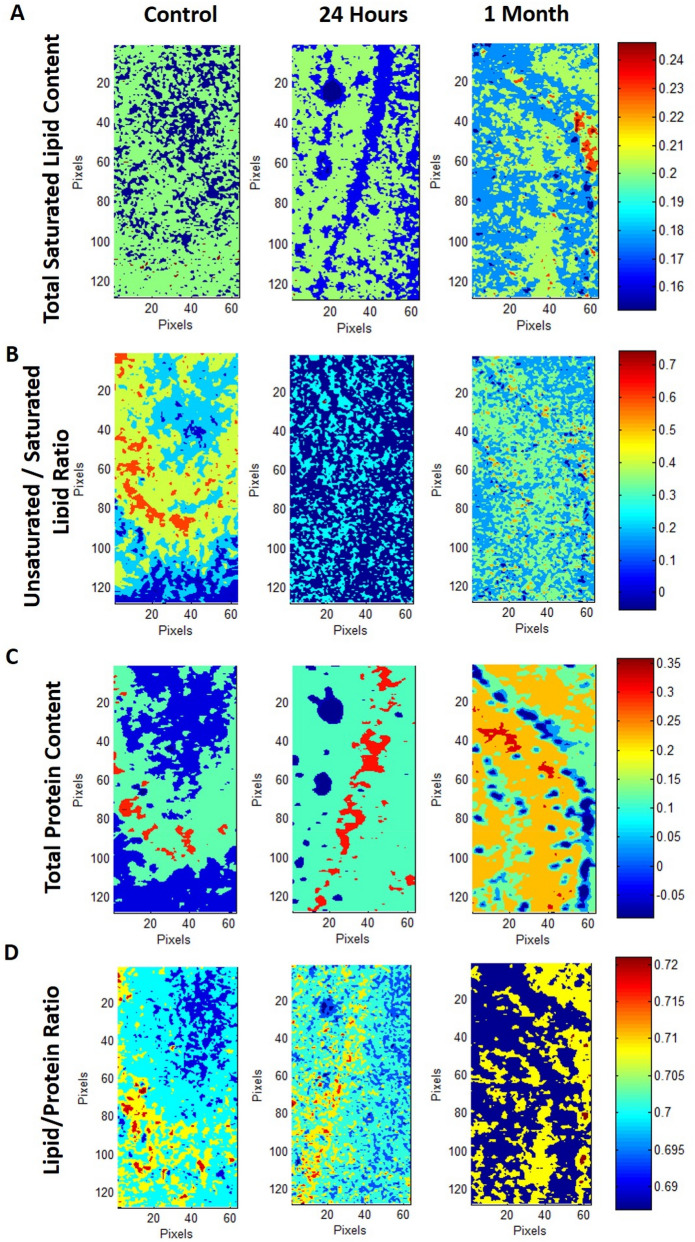
Typical FTIR images of total saturated lipid content (**A**), unsaturated/saturated lipid (**B**), total protein content (**C**), lipid/protein band area ratios in right hippocampal tissue of control, 24 h and 1-month TBI groups. Color bars represent the scales for each of the parameters. Axes are in pixels, where one pixel is 6.5 μm.

To obtain more qualitative information about lipids, such as carbonyl group content and unsaturated fatty acid content in tissue lipids, carbonyl/lipid and unsaturated/saturated lipid band area ratios were calculated (Fig. 2). The carbonyl to lipid ratio, implying the amount of carbonyl groups in lipid structures in the membranes^16^, was calculated by taking the band area ratios of the C=O ester stretching band (1717–1759 cm^−1^) to the C–H region (2988–2832 cm^−1^). Moreover, the unsaturated to saturated lipid ratio, implying the unsaturation index^25^ in tissues was calculated by taking the band area ratio of the olefinic band (3024–3000 cm^−1^: unsaturated lipid) to the C–H region (total saturated lipid). The olefinic band, which originates from unsaturated HC=CH vibrations in the lipids of the tissue, can be used to measure lipid peroxidation^33^. As seen from Figs. 2 and 5, both carbonyl/lipid and unsaturated/saturated lipid ratios were significantly decreased in the 24 h TBI group when compared to the control group, whereas in the 1-month TBI group these band ratios were (partially) normalized (Fig. 2). From the pixel histograms, which are shown in Fig. 3, it can be clearly seen that the pixels were shifted to left side of the histogram implying a decrease in the ratio for the 24-h group compared to the control group, whilst the pixels were shifted to the right side (close to the position of the pixels in the control group) in the 1-month TBI group (Fig. 3).

In the hydrocarbon chains of the lipids, CH_2_ functional groups were located at the trunk portion, while CH_3_ functional groups were placed at the tip portion. Thus, the ratio of the areas of the CH_2_ anti-symmetric stretching band to that of the CH_3_ anti-symmetric stretching band (2988–2944 cm^−1^) gives information about the qualitative lipid hydrocarbon chain length^33^. In our study, lipid acyl chain length was significantly decreased at 24 h after TBI when compared to the control group, while there was no change at 1-month after TBI, as seen from Figs. 2 and 4. When we determined the acute and chronic molecular changes after TBI in terms of structure and acyl chain length of lipids in the hippocampus, significant alterations were seen at 24 h after the injury, while normalization of these parameters was seen 1-month after TBI. This finding was supported by the pixel histograms, in which the pixels were moved to the left side of the histogram, implying lower ratio values in the 24-h TBI group, while there was no change in the 1-month TBI group when compared to the control group (Fig. 3). Although the value of the ratio did not change in the 1-month TBI group, a broadened pixel distribution (higher heterogeneity of pixels) was observed as compared to the control group.

It is known that the band positions of the CH_2_ anti-symmetric and symmetric stretching bands can be used to determine the conformational disorder of the lipids and to examine the average trans/gauche isomerization in the system^34^. Furthermore, the bandwidth of the mentioned bands gives information about fluidity and dynamics of the membrane^34,35^. The shift of the peak position of CH_2_ symmetric stretching band to higher wavenumber value implies an increase in gauche conformers e.g. increase in disorder of acyl chains, in other words increase in acyl chain flexibility in the membrane lipids of hippocampal tissue in the 24-h TBI group compared to the control^16,34^ (Table 2). Moreover, an increase in bandwidth values of CH_2_ symmetric stretching band in the 24-h TBI group indicates an increase in membrane fluidity of hippocampal tissue compared to the control group (Table 2). However, there was no alteration, neither in wavenumber nor in the bandwidth values of CH_2_ symmetric stretching band, in the 1-month TBI group (Table 2). These results were supported by the wavenumber and bandwidth values of the CH_2_ anti-symmetric stretching band in the TBI groups (data not shown).

**Table 2:** The wavenumber and bandwidth values of CH_2_ symmetric stretching band of control, 24 h and 1 month rat groups.

		p value
**CH**_**2**_** symmetric stretching band wavenumber value**		
Control	2852 ± 0.2	–
24 h	2853 ± 0.3	≤ 0.05
1 month	2852 ± 0.5	NS
**CH**_**2**_** symmetric stretching bandwidth value**		
Control	0.5 ± 0.01	–
24 h	0.6 ± 0.02	≤ 0.05
1 month	0.5 ± 0.04	NS

To determine the acute and chronic effects of TBI on protein structure and content, amide I (1672–1636 cm^−1^) and amide II (1560–1536 cm^−1^) bands, which are two major protein bands in FTIR, were examined. Total protein content was measured by calculating the ratio of areas under the amide I band and amide I + amide II band. We found that total protein content was decreased in the 24-h TBI group, while there was an increase in the 1-month TBI group when compared to the control group (Figs. 2, 4). This result was supported by the pixel histograms, in which the pixels were moved to the left side of the histogram, implying lower ratio values in the 24-h TBI group, while there was a shift to the right side of the histogram with higher ratio values in the 1-month TBI group when compared to the control group (Fig. 3).

The lipid to protein ratio, which was calculated by taking the ratio of the integrated areas of the C–H stretching region (total lipid) to the integrated areas of amide I + amide II (total protein) bands, was used to measure the relative amount of proteins and lipids in the system. This ratio is an important parameter in molecular asymmetry^25,36^ and has an important effect on membrane structure and dynamics^35,37^. As seen from Figs. 2 and 4, there was a decrease in lipid/protein ratio after TBI, especially at the chronic stage, compared to the control group. The decrease in this ratio was also evident from the pixel histograms of the groups (Fig. 3). However, this ratio may be obscured when similar degrees of variation in lipid and protein content occur.

Figure 1S shows the protein and lipid distributions separately on the raw absorption maps in control, 24 h and 1-month post TBI groups to determine the possible spatial/reorganization effect of injury on hippocampus. To get the information about the distribution of proteins, Amide I band was used as a reference and for the distribution of saturated lipids CH_2_ asymmetric stretching band was used. As seen from the Fig. 1S, in control group there is a limited distribution of both proteins and lipids on the absorption maps, while after TBI there is a wider distribution of these biomolecules on the absorption map of 1-month post TBI group compared to both control and 24 h post TBI groups.

Amide I and Amide II bands can be used to determine changes in protein structure, since they are sensitive to changes in protein conformation^38^. For this aim, the band area ratios of amide I/amide II were calculated to get information about the changes in protein structure^35,39^. Amide I to amide II ratio significantly decreased in the 24-h and 1-month TBI groups, revealing a significant change in protein secondary structure when compared to the control group (Figs. 2, 4). This decrease was also evident from the pixel histograms, which were shifted to the left side of the histogram when compared to the control group histogram (Fig. 3).

Metabolic activity of the tissue (glycogen/phosphate ratio) can be measured by calculating the ratio of the area of the νC–O + δC–O stretching band (1049 cm^−1^) to the area of the PO_2_^−^ symmetrical stretching band (1100–1060 cm^−1^)^40^. The νC–O + δC–O stretching band is due to glycogen and carbohydrate molecules in the system^40^, while the PO_2_^−^ symmetrical stretching band originates mainly from nucleic acids with a contribution from head groups of phospholipids in membranes^40^. As seen from Figs. 2 and 4, the glycogen/phosphate ratio was significantly decreased in the 24-h TBI group while there was no change in the 1-month TBI group compared to the control group. This result was supported by the pixel histograms of the 24-h and 1-month TBI groups, in which pixels were shifted to the left and right side of the histogram, respectively (Fig. 3). This result reveals an acute effect of TBI on the metabolic activity of hippocampus followed by recovery of the activity at one month after the injury. As seen from Fig. 3, broadened pixel distributions (higher heterogeneity of pixels) were also seen in the 24-h and 1-month TBI groups compared to the control.

Figure 5 shows the representative absorption maps for calculated peak area ratios including total protein (Amide I to Amide I + Amide II) and saturated lipid content (CH_2_ asym stretching to C–H region), lipid/protein ratio and the amount of unsaturated to saturated lipid ratio (olefinic band to C–H region) in right hippocampus tissue. The acute and chronic molecular changes in right hippocampus tissue was similar to the ones seen in left hippocampus including significant gradual increase in saturated lipid content, decrease in lipid to protein ratio in both 24-h and 1-month post TBI groups while a significant decrease in unsaturated to saturated lipid ratio in 24 h after TBI and a partial return to the control group levels in 1 month after TBI (Fig. 5). The changes in the peak area ratios of carbonyl to lipid and Amide I to Amide II in right hippocampus tissue were also similar to the left hippocampus (data not shown). The only difference from the results of left hippocampus was seen in total protein content in 24 h after TBI in right hippocampus. In total protein amount, as seen from Fig. 5, there was a significant gradual increase in both 24 h- and 1-month post TBI groups compared to the control which was a different result than left hippocampus since there was a decrease in this ratio in 24-h post TBI groups and a significant elevation in 1-month post TBI group in left hippocampus tissue. Shifting to higher wavenumber values for the CH_2_ symmetric stretching band (increase in acyl chain flexibility in the membrane lipids) with an increase in bandwidth values of CH_2_ symmetric stretching band (increase in membrane fluidity) were also seen in right hippocampus tissue of 24-h post TBI group while there was no alteration in 1-month post TBI group compared to the control (data not known).

## Discussion

In the current study, the molecular effects of mild traumatic brain injury (TBI) on the hippocampus of rat brain tissue were investigated at 24 h and 1 month after the injury to determine the acute and chronic effects, respectively. FTIRM was used to examine the structural and contextual alterations of biomolecules including lipids, proteins, and carbohydrates in hippocampal tissue.

Mild traumatic brain injury, experimentally induced by weight drop, resulted in changes in the content and structure of many biomolecules in the hippocampus 24 h after injury. For most of these early TBI-induced alterations, there was a recovery effect at one month after the injury. The acute and chronic molecular changes in both right and left hippocampus tissues were similar except the increase in total protein content in 24-h post TBI group in the right hippocampus.

The involvement of lipid metabolism in neuronal degeneration and repair is well-known^5^. It was previously reported that lipid peroxidation occurs after severe traumatic brain injury in humans and correlates with the injury severity and mortality^41^. Acidic conditions seen after TBI induce a disbalance between O_2_^−^ and HO_2_ leading to lipid peroxidation^2,42^, which is associated with both mitochondrial dysfunction and cytoskeletal degradation in vivo^20,43^. In lipid peroxidation, spontaneous and enzymatic conversion of O_2_^−^ forms reactive oxygen species (ROS), to which the brain tissue is highly sensitive due to its high O_2_ consumption, concentration of redox transition metals and concentration of polyunsaturated fatty acids (PUFAs)^44^. Since lipid peroxidation mainly occurs at double-bond sites (C=C–C and C=O) of PUFAs, the significant reduction in the amount of unsaturated lipids is due to loss of olefinic bonds^25^. In the current study, there was a significant decrease in olefinic = CH/lipid ratio, which gives information about the unsaturation level of the system, in the 24-h TBI group implying lipid peroxidation in the hippocampus as a consequence of TBI. However, there was no significant difference between the 1-month TBI group and the control group, which suggests an improvement in lipid metabolism in hippocampal tissue chronically after mild TBI.

In previous studies, Cakmak et al.^25^ and Elibol et al.^26^ also reported decreased olefinic/lipid ratios in FTIR studies resulting from increased lipid peroxidation due to whole body ionizing radiation on rat brain homogenate membranes and vitamin A deficiency in hippocampal tissue, respectively. However, Zhang et al.^20^, who investigated the effects of traumatic axonal injury (TAI) on the corpus callosum (CC) in rat brain tissues at 12, 24, and 72 h post-injury, showed increased unsaturation levels in the CC. They suggested that loss of unsaturation due to lipid peroxidation was compensated by the accumulation of double bonds in lipid peroxidation end-products, such as lipid aldehydes and alkyl radicals^20^. These conflicting results may be due to the difference in injury types, and different content and composition of particular regions of the studied brain tissues. The decrease in this ratio can also be explained by the increase in total saturated lipid amount. The elevation in total saturated lipid content besides the decrease in unsaturated lipid content in the hippocampus of chronic post-TBI groups were also reported in a previous study^45^.

Besides the reduction in unsaturated lipid content, there was a significant decrease in carbonyl content and lipid hydrocarbon chain length in the 24-h TBI group. These results may also be due to the breakdown of membrane lipids, which leads to shortened fatty acid chain lengths, and consequently altered lipid composition and distribution^25^ in the hippocampus as an acute effect of TBI. The variations in lipid content may result in changes in lipid asymmetry which means an imbalance in the distribution of the inner and outer leaflets of the membrane bilayer^25,46^. Our results are in agreement with previous studies that reported the occurrence of lipid peroxidation in brain tissue after traumatic axonal injury (TAI)^20^, intracerebral hemorrhage^24^ and diffuse axonal injury (DAI)^21^. Moreover, there was a slight decrease in saturated lipid content and no change in lipid acyl chain length in the 1-month TBI group as compared to the control group, implying a recovery effect on lipid composition chronically after TBI.

Changes in content and composition of lipids, including variations in unsaturation level and acyl chain length, and changes in protein content and their lateral distribution in membranes are responsible for the membrane order^47^. The order information obtained from the wavenumber values of the CH_2_ symmetric stretching band in FTIR studies gives information about the changes in the trans:gauche ratio in acyl chains^26^. In the current study, higher wavenumber values of this band in hippocampal tissue of the 24-h TBI group indicates higher acyl chain flexibility implying a decrease in lipid order after TBI^47^. There was no change in the wavenumber value of this band in the 1-month TBI group compared to the control group, which indicates recovery in membrane lipid order state chronically after mild TBI. It was previously reported that membrane thickness decreases with the decrease in lipid order^25,48^. Accompanying changes in membrane asymmetry and membrane thickness with the lipid peroxidation may induce alterations in the membrane potential via ion channel kinetics^49^ resulting in membrane damage, eventual apoptosis and finally tissue necrosis^2^. The current study also showed that TBI can cause an increase in membrane fluidity in the 24-h TBI group compared to the control group. Higher membrane fluidity induced by brain injury may be caused by the difference in membrane lipid packing mentioned before. However, similar as for lipid order, there was no alteration in membrane fluidity one month after TBI, implying an improvement in membrane fluidity and order, which may contribute to recovery of brain tissue functioning.

Another critical parameter that affects membrane structure and function is the amount of lipids and proteins in biological membranes^25^. Changes in the lipid to protein ratio imply variation in the lipid and protein metabolism in brain tissue after TBI. This ratio also gives information about the changes in the lipid and protein asymmetry in bilayer^48^ that is closely related to membrane function as discussed above. In the present study, this ratio was slightly increased in the 24-h TBI group and significantly decreased in the 1-month TBI group compared to the control group. The latter could be attributed to a more notable increase in protein content since we observed a significant increase in both lipid and protein content in the 1-month TBI group. This result was also supported by the elevation of total protein content in this group. Williamson et al.^24^, who assessed the elemental and biochemical effects of rehabilitation after intracerebral hemorrhage using FTIR Imaging, reported increased lipid content in the peri-hematoma zone following rehabilitation. This study is in line with our finding of increased hippocampal lipid content in recovering rats at 1-month after mild TBI. Moreover, in the study of Abdullah et al.^45^, it was reported that hippocampal phosphatidylcholine (PC), phosphatidylethanolamine (PE) and sphingomyelin levels were elevated significantly in chronic post TBI mice. This study supports the results of the current study in which there was an elevation in total lipid content in 1-month post TBI animal group with a wide distribution of lipids in the absorption map compared to the control group.

In an earlier study, the time course of synapse replacement was mapped based on structural, molecular and functional parameters in the hippocampal dentate gyrus. In this study, it was reported that synaptic recovery was preceded by early degeneration between 1 and 6 days and followed by subsequent regeneration at 7–21 days after traumatic brain and spinal cord injuries^50^. Time-specific metalloproteinase (MMP) signaling in the postinjury state may be critical for synaptic recovery after both traumatic brain and spinal cord injuries^51^. Members of the MMP family were shown to exhibit an axon degeneration–synapse regeneration cycle, which is initiated over the first 2 weeks after injury^49^. Although there is an induction of quite a number of MMPs postinjury, MMP-3 and ADAM thrombospondin (TS) upregulation appear to play a main role at onset of regenerative processes^51^. The time-dependent changes in the expression and induction of these proteins in brain tissue after TBI are in line with our results including the reduction in total protein content at 24 h after injury (just in left hippocampus) and the elevation of this content one month after injury (both in left and right hippocampus), reflective of the axon degeneration–synapse regeneration cycle.

In the current study, spatial/reorganization effect of injury on hippocampus can also be seen by the limited distribution of both proteins and lipids on the absorption maps of control, while a wider distribution of these biomolecules on the absorption map of 1-month post TBI group compared to both control and 24 h post TBI groups. It is a consequence of the recovery process in chronic stage of post TBI in the hippocampus tissue. Adult neurogenesis has been shown to primarily occur in only subventricular zone and the subgranular zone of the hippocampus^52^. Adult hippocampal radial-glia like (RGL) progenitor cells found in the subgranular zone of hippocampus, produce daughter neuroblasts which migrate to the dentate gyrus sunfield. Then in their final destination they differentiate into immature neurons and over time become mature neurons^52^. This explains the site-specific or limited protein and lipid distribution in the absorption maps of the hippocampus of control group in the current study. Although moderate-severe TBI has been shown to trigger acute loss of newborn neurons besides hyper-proliferation of progenitor cell in rodents, there is no report about the alteration of hippocampal neurogenesis in mild TBI^53^. However, there are several studies showing site-specific up-regulation of non-neuronal genes which codes transcription factors such as Nuclear factor erythoid related factor 2 (NRF2), growth factors [insulin like growth factor 1 (IGF-1)] with a coincident increase in several proteins (heme-oxygenase-1, nicotinamide adenine dinucleotide phosphate-quinone-oxidoreductase 1, glutathione reductase, and catalase) in post-TBI hippocampus after 48–72 h and 1 week^52^. This explains the increased amount and wide distribution of proteins in 1-month TBI group in the current study. The start of increase in the protein amount in right hippocampus at 24 h after injury but not in left hippocampus, may be explained by the early expression of those proteins in the acute stage just after TBI in right hippocampus and over time in left hippocampus.

Furthermore, the band area ratio of amide I to amide II significantly decreased in both TBI groups compared to the control group implying some alterations in the structures of proteins in hippocampal tissue. The axon degeneration–synapse regeneration cycle through the induction of MMP proteins may explain these structural changes reflected by the alteration of the expression of target proteins in acute and chronic states of mild TBI. In another study, Zhang et al.^54^ investigated amide I band with curve fitting to determine the secondary structural changes of proteins due to axonal injury in the brainstem of rats 72 h after TBI. The authors reported that the significant protein conformational changes in injured axons compared to the normal ones may indicate the potential of amide I to be an infrared spectral marker of axonal injury.

The brain is sensitive to glucose and oxygen deprivation due to its high energy consumption. Hyper- and hypoglycolysis conditions are important contributors to the effects of TBI following initial injury^20^. Lou et al.^55^ reported that hyperglycolysis occurs in the first hours up to 5 days after injury due to disruption of the blood–brain barrier (BBB) and increased glucose uptake through another mechanism than glucose transporters in the membrane. In the current study, glycogen to phosphate ratio was significantly decreased in the 24-h TBI group implying a hyperglycolysis state in initial post-injury resulting in a decrease in glycogen and carbohydrate content. There was no change in this parameter in the 1-month TBI group compared to the control group. The latter result suggests an improvement in metabolic activity of the hippocampus, possibly through regulation of the carbohydrate level chronically after TBI. Zhang et al.^20^ suggested the use of time-dependent spectral changes regarding carbohydrates in CC as a potential parameter for the estimation of traumatic axonal injury intervals.

In the current study, for some calculated parameters a broadened pixel distribution (higher heterogeneity of pixels) was observed without any change in value as compared to the control group. In previous studies, it was reported that the changes in pixel distribution of FTIR imaging parameters were observed due to the associated effects of age and/or some diseases in tissues pathology^56^.

In the previous studies, it was shown that TBI can lead to temporary or permanent cognitive impairment including attention deficits and poor executive functioning, as well as sensorimotor deficits, and behavioral problems^57,58^. Damage to the hippocampus after TBI may be responsible for impairment of spatial learning and memory^59^. In another study of our group^58^, behavioural assessment with two well established tests [sensorimotor deficit score (SDS) and Beam walk test] and histological examination were carried out in 24-h and 3–4 months post-mild and -moderate TBI animals to get information about sensorimotor function and microstructural tissue status, respectively. Differences in the myelin density of the control and post-TBI groups were assessed by myelin basic protein (MBP) staining in this study. It was reported that although no significant differences were found among control, mild TBI, and moderate TBI rats in myelin density, at 24 h post-TBI, all regions showed trends of decreasing myelin density in both mild and moderate TBI groups. It was also shown that after 3–4 months, myelin density in TBI groups increased compared to the 24 h post TBI groups. The recovery could be started after 1-month as seen in our current results but there is no evidence for that since the myelin density was not measured in 1-month post-TBI group animals in that study. Moreover, in the same study it was reported that sensorimotor performance was significantly affected after moderate TBI, but not in any post mild TBI animal groups according to the behavioural tests^58^.

Some other studies reported correlations between the molecular changes in white matter region of brain and behavioural deficits^60,61^. However, in these studies different brain damage model, a controlled cortical impact model (including more focal and lateralized brain damage) was used instead of our acceleration/deceleration model. In another study^62^ which used a weight drop model similar to our model, motor deficits and cognitive deficits associated with the injury were measured using beam walk and Morris water maze tests, respectively in acute (24 h after the injury) and chronic (7 and 21 days after the injury) mild TBI phases. The Morris water maze test has proven to be extremely valuable for distinguishing performance based on spatial learning and memory related to the hippocampus in animals^59^. In the study of Heffernan et al. (2013), it was reported that there was no significant differences in motor deficits between groups, while the Morris water maze test indicated that cognitive deficits persisted for the first week after injury and recovered in the chronic phases after 3 weeks^62^. We have no cognitive performance data neither in the current study, nor in our previous study mentioned above^58^. However, the results of the cognitive tests of Heffernan et al. (2013) were in line with our current dramatic molecular changes in acute phase (24 h post TBI) and recovery in the chronic phase (1-month post TBI).

## Conclusion

Mild TBI can cause significant structural and contextual changes in hippocampal tissue, including decrease in carbonyl content, unsaturated lipid content, lipid acyl chain length, membrane lipid order, total protein content and lipid/protein ratio, besides increase in membrane fluidity with an altered protein structure and metabolic activity at 24 h after injury. However, a recovery effect and an improvement in these parameters was found to occur at one month after TBI.

A limitation of the study is the limited number of time-points we investigated after TBI, i.e. 24-h and one month post-injury. More (earlier, intermediate and later) time points could help to further elucidate the temporal effects of TBI. Another limitation is that effects were only measured in the hippocampus. Investigating different brain regions may reveal possible differences in mechanisms of injury and recovery after TBI. Moreover, there is no sham injury group in our study. The presence of this animal group would have allowed assessment of possible effects of the animal preparation on the outcome measures.

## Materials and methods

Animal procedures were conducted according to guidelines of the European Communities Council Directive and approved by the local Ethical Committee on Animal Experiments of the Utrecht University and University Medical Centre Utrecht. All experimental protocols and methods in this study were approved by University Medical Centre Utrecht. Moreover, animal procedures and experimental protocols adhered to the ARRIVE (Animal Research: Reporting of In Vivo Experiments) guidelines.

### Traumatic brain injury (TBI) animal model

In the current study, 11-week-old male Sprague Dawley (= SD) (Charles River Laboratories International, Wilmington, MA, USA) rats (350–400 g) were allocated into three different groups, i.e. controls (n = 5), 24-h post-TBI (n = 5) or 1-month post-TBI (n = 4). Rats were kept at room temperature under a constant 12-h light/dark cycle with food and water ad libitum. A mild form of TBI was induced by weight drop as described previously^63,64^. Rats were first anaesthetized with 4% isoflurane for endotracheal intubation, followed by mechanical ventilation with 2.0–2.5% isoflurane in air/O_2_ (4:1) (Sigma, Netherlands). Rats received a subcutaneous injection of 0.05 mg/kg buprenorphine (Temgesic, Schering-Plough, Netherlands) for postsurgical pain relief. A weight of 450 g was dropped from 1-m height through a Plexiglas tube, on to the center of a steel disc, which was temporally glued to the skull midline of rats between bregma and lambda. The animal’s head was supported by a foam bed during the procedure. The steel disk prevented the skull from fracturing and distributed the impact power over a larger brain area. The animals were returned to their cages after the incision was stitched and they regained complete consciousness. Control animals were anesthetized and underwent all the surgical procedures except the weight drop.

Rats received an overdose of isoflurane followed by transcardial perfusion with 4% paraformaldehyde (PFA) in phosphate-buffered saline at 24-h (for the control and 24 h groups) and 1-month (for the one-month group) after (sham) TBI induction. The brains were removed and post-fixed in 4% PFA at 4 °C for 24 h, followed by embedding in paraffin.

### Sample preparation for histology and FTIR imaging studies

10-μm thick two adjacent brain tissue sections were cut using a soft-tissue microtome (Leica RM 2155 semi-automated rotary microtome, Germany). One section was used for histology and the other adcajent section was used for FTIR Imaging studies.

For H&E staining, the sections were first dehydrated using alcohol, stained with hematoxylin and soaked in eosin. Then the slices were soaked into gradient concentrations of alcohol for dehydration, cleared with xylene, and finally sealed as described in our previous studies^28,29^. Stained sections were assessed with a light microscope under 40× magnification (Fig. 1A).

For FTIR Imaging studies, 10 μm sections were directly transferred onto IR transparent barium fluoride windows (Spectral Systems, NY, USA). These IR sections were kept in a desiccator with a vacuum pump in a cold room overnight to remove the moisture from the sections.

### Data collection for FTIR imaging and data analysis

FTIR images were recorded using an Agilent FTIR Cary 620 micro-spectrometer (Agilent, Santa Clara, USA) in transmission mode at a spectral resolution of 4 cm^−1^ within the wavenumber range of 4000–700 cm^−1^ with a 5.5 × 5.5 μm^2^ pixel size of 64 × 64 Mercury Cadmium Telluride (MCT) detector and 32 scan numbers per pixel. In each brain sample three different areas from the left and right hippocampus were randomly chosen to collect IR maps by scanning the chosen areas pixel by pixel (pixel size: 6.25 × 6.25 μm^2^) (Fig. 1B,C) and getting an IR spectrum from each pixel. A total of 7200 spectra were recorded from each chosen area (374 × 750 μm^2^) of each section.

The background spectra were recorded from the empty IR window and automatically subtracted from the collected spectra of the brain tissue by using Resolution Pro. Software (version 5.0, Agilent Technologies, Santa Clara, CA, USA).

Since the differences in section thickness may result in concentration-dependent spectral changes in absorbance values, band area and/or band intensity ratios were used for the calculation of contextual and structural parameters to avoid possible errors due to section thickness in FTIR image analysis^32^. The imaging results were expressed as representative color-coded images and histograms, which describe the pixel distribution of the parameters above, as mean values and standard deviations of the pixel distributions (Fig. 1E). Moreover, spectral parameters including lipid order and membrane fluidity were determined from band frequency and bandwidth values of special bands. Spectral images were analyzed using ISYS software (Version 2.1, Spectral Dimensions, Olney, MD, USA, https://www.pharmamanufacturing.com/vendors/products/2005/293/).

### Statistical analysis

Statistical analysis was performed using GraphPad Prism (Version 8.1.2, GraphPad Software, San Diego, CA, USA, https://www.graphpad.com/scientific-software/prism/) software. After testing data for normal distribution and homogeneity of variance, the FTIR results for each group, expressed as colour-coded images and pixel population means, were statistically analyzed with one-way ANOVA with Tukey’s post hoc test. The coefficient of variation (standard deviation/mean) was calculated for each parameter in each animal and the data were summarized as the mean and standard deviations for each group. p values less than or equal to 0.05 were considered as statistically significant for comparisons *p ≤ 0.05; **p ≤ 0.01, ***p ≤ 0.001).

## Supplementary Information

Supplementary Information 1.

Supplementary Information 2.
