# Supplementary material for: Biomolecular changes and subsequent time-dependent recovery in hippocampal tissue after experimental mild traumatic brain injury
Source: Sci Rep. 2021 Jun 14;11:12468. doi: 10.1038/s41598-021-92015-3 (PMC8203626; doi:10.1038/s41598-021-92015-3)
Supplement: Supplementary file 1 — Supplementary Information 1. [file 41598_2021_92015_MOESM1_ESM.docx]

**Biomolecular changes and subsequent time-dependent recovery in hippocampal tissue after experimental mild traumatic brain injury**

**Sebnem Garip Ustaoglu^1*^,** **Mohamed H. M. Ali^2*^, Fazle Rakib^3^, Erwin L.A. Blezer^4^, Caroline L. van Heijningen^4^, Rick M. Dijkhuizen^4^, Feride Severcan^5,6^**

**Affiliations:**

1. Department of Medical Biochemistry, Faculty of Medicine, Altinbas University, Bakirkoy, Istanbul, Turkey
2. Diabetes Research Center, Qatar Biomedical Research Institute (QBRI), Hamad Bin Khalifa University (HBKU), Qatar Foundation (QF), P.O. Box 34110 Doha, Qatar.
3. Department of Chemistry and Earth Sciences, Qatar University, Doha, Qatar
4. Biomedical MR Imaging and Spectroscopy Group, Center for Image Sciences, University Medical Center Utrecht, Utrecht, The Netherlands.
5. Department of Biophysics, Faculty of Medicine, Altinbas University, Bakirkoy, Istanbul, Turkey
6. Department of Biological Sciences, Middle East Technical University, Ankara, Turkey

*Correspondence to:

**Sebnem Garip Ustaoglu**

Medical Biochemistry Department

Faculty of Medicine, Altinbas University

E-mail: [sebnem.garip@altinbas.edu.tr](mailto:sebnem.garip@altinbas.edu.tr)

Phone: +90 212 709 4528 Ext.5246

**Mohamed H.M. Ali**

Diabetes Research Center, Qatar Biomedical Research Institute

Hamad Bin Khalifa University, Qatar Foundation

Email: mohamali@hbku.edu.qa

**Table 1S:** The integrated spectral ranges of bands used in the analysis.

| **Infrared Band** | **Integrated Spectral Range (cm^-1^)** |
| --- | --- |
| Olefinic=CH | 3024-3000 |
| CH_3_ anti-symmetric stretching | 2988-2944 |
| CH_2_ anti-symmetric stretching | 2944-2896 |
| CH_2_ symmetric stretching | 2832-2863 |
| C-H stretching | 2988-2832 |
| Carbonyl ester (C=O) stretching | 1717-1759 |
| Amide I | 1672-1636 |
| Amide II | 1560-1536 |
| PO_2_^-^ symmetric stretching | 1100-1060 |
| νC-O + δC-O stretching | 1060-1000 |
