# Supplementary material for: Biomolecular changes and subsequent time-dependent recovery in hippocampal tissue after experimental mild traumatic brain injury
Source: Sci Rep. 2021 Jun 14;11:12468. doi: 10.1038/s41598-021-92015-3 (PMC8203626; doi:10.1038/s41598-021-92015-3)
Supplement: Supplementary file 2 — Supplementary Information 2. [file 41598_2021_92015_MOESM2_ESM.docx]

**Biomolecular changes and subsequent time-dependent recovery in hippocampal tissue after experimental mild traumatic brain injury**

**Sebnem Garip Ustaoglu^1*^,** **Mohamed H. M. Ali^2*^, Fazle Rakib^3^, Erwin L.A. Blezer^4^, Caroline L. Van Heijningen^4^, Rick M. Dijkhuizen^4^, Feride Severcan^5,6^**

**Affiliations:**

1. Department of Medical Biochemistry, Faculty of Medicine, Altinbas University, Bakirkoy, Istanbul, Turkey
2. Diabetes Research Center, Qatar Biomedical Research Institute (QBRI), Hamad Bin Khalifa University (HBKU), Qatar Foundation (QF), P.O. Box 34110 Doha, Qatar.
3. Department of Chemistry and Earth Sciences, Qatar University, Doha, Qatar
4. Biomedical MR Imaging and Spectroscopy Group, Center for Image Sciences, University Medical Center Utrecht, Utrecht, The Netherlands.
5. Department of Biophysics, Faculty of Medicine, Altinbas University, Bakirkoy, Istanbul, Turkey
6. Department of Biological Sciences, Middle East Technical University, Ankara, Turkey

*Correspondence to:

**Sebnem Garip Ustaoglu**

Medical Biochemistry Department

Faculty of Medicine, Altinbas University

E-mail: [sebnem.garip@altinbas.edu.tr](mailto:sebnem.garip@altinbas.edu.tr)

Phone: +90 212 709 4528 Ext.5246

**Mohamed H.M. Ali**

Diabetes Research Center, Qatar Biomedical Research Institute

Hamad Bin Khalifa University, Qatar Foundation

Email: mohamali@hbku.edu.qa


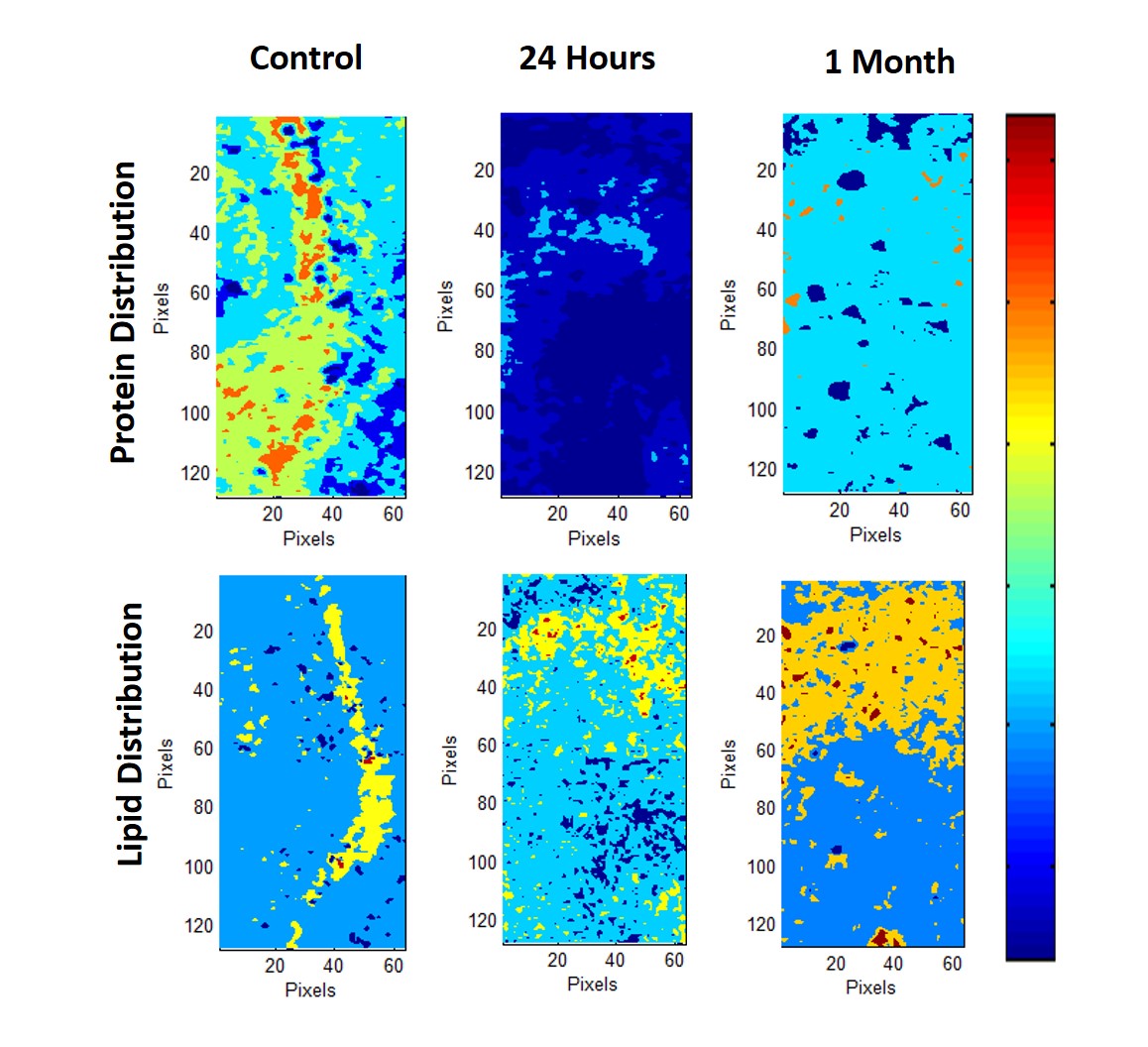


**Figure 1S.** Protein and lipid distribution in left hippocampal tissue of control, 24h and 1-month TBI groups. Protein and lipid distribution were obtained by using integrated areas of Amide I band and CH2 asymmetric stretching band respectively in the raw absorption maps of the groups. Color bars represent the highest (dark red) and lowest (dark blue) scales for the absorption maps. Axes are in pixels, where one pixel is 6.5 μm.
